# Carbapenem Resistance among *Enterobacter* Species in a Tertiary Care Hospital in Central India

**DOI:** 10.1155/2014/972646

**Published:** 2014-08-10

**Authors:** Atul Khajuria, Ashok Kumar Praharaj, Mahadevan Kumar, Naveen Grover

**Affiliations:** Department of Microbiology, Armed Forces Medical College, Pune 411040, India

## Abstract

*Objective*. To detect genes encoding carbapenem resistance among *Enterobacter* species in a tertiary care hospital in central India. *Methods*. Bacterial identification of *Enterobacter* spp. isolates from various clinical specimens in patients admitted to intensive care units was performed by routine conventional microbial culture and biochemical tests using standard recommended techniques. Antibiotic sensitivity test was performed by standard Kirby Bauer disc diffusion technique. PCR amplification and automated sequencing was carried out. Transfer of resistance genes was determined by conjugation. *Results*. A total of 70/130 (53.84%) isolates of *Enterobacter* spp. were found to exhibit reduced susceptibility to imipenem (diameter of zones of inhibition ≤13 mm) by disc diffusion method. Among 70 isolates tested, 48 (68.57%) isolates showed MIC values for imipenem and meropenem ranging from 32 to 64 mg/L as per CLSI breakpoints. All of these 70 isolates were found susceptible to colistin *in vitro* as per MIC breakpoints (<0.5 mg/L). PCR carried out on these 48 MBL (IP/IPI) *E*-test positive isolates (12 *Enterobacter aerogenes*, 31 *Enterobacter cloacae*, and 05 *Enterobacter cloacae* complex) was validated by sequencing for beta-lactam resistance genes and result was interpreted accordingly. *Conclusion*. The study showed MBL production as an important mechanism in carbapenem resistance in *Enterobacter* spp. and interspecies transfer of these genes through plasmids suggesting early detection by molecular methods.

## 1. Introduction

Beta-lactams are one of the most frequently used classes of antimicrobials in hospital settings, crucial for the treatment of infections caused by Gram-negative bacteria.* Enterobacter* spp. are common pathogens of Enterobacteriaceae family responsible for nosocomial infections, especially blood stream infections in intensive care units.* Enterobacter* may produce severe diseases including those of abdomen, lower respiratory tract, urinary tract, meningeal, eye, bone, and surgical site infections [1]. As per National Nosocomial Infection Surveillance System, more than one-third of the* Enterobacter* spp. are resistant to extended-spectrum cephalosporins in intensive care units [2]. However, of late due to the presence of extended-spectrum beta-lactamase (ESBL) and AmpC enzymes in* Enterobacter* spp., Carbapenems have become the drug of choice to treat such infections [3]. There has been an increase in incidence of multidrug resistance in these organisms due to dissemination of resistance determinant genes mediated by transposons, plasmids, and gene cassettes in integrons. To understand the widespread occurrence of the beta-lactamases in* Enterobacter* spp., we conducted a study to detect beta-lactam resistance genes along with plasmid replicon typing of carbapenem resistant* Enterobacter* spp. isolates recovered from clinical specimens in a tertiary care hospital in central India.

## 2. Materials and Methods

### 2.1. The Bacterial Isolates

A prospective study was conducted in a 1000 bedded tertiary care centre in Pune, India, from October 2011 to May 2013. A total of 130* Enterobacter* spp. isolates (45* Enterobacter aerogenes*, 62* Enterobacter cloacae*, and 23* Enterobacter cloacae* complex) were recovered from clinical specimens from different patients (one isolate per patient) admitted to the medical and surgical intensive care units. Collection of sample was done using strict aseptic precautions and was immediately processed without any delay. The isolates were obtained from various clinical specimens such as cerebrospinal fluid, bone marrow, blood, pus, urine, lower respiratory secretions (endotracheal secretions, bronchoalveolar lavage, and bronchial wash), sputum, tissues, and other sterile body fluids. Bacterial identification was performed by routine conventional microbial culture and biochemical tests using standard recommended techniques [4]. The organisms were identified up to the species level using VITEK-GNI cards (bioMérieux, Marcy l'Etoile, France).

### 2.2. Antimicrobial Susceptibility Testing

The antimicrobial susceptibility was performed by the Kirby Bauer's disc diffusion technique on Mueller-Hinton agar, as per Clinical Laboratory Standard Institute (CLSI) guidelines [5]. The antibiotics tested were as follows (potency in *μ*g/disc): Ampicillin (10), Cefuroxime (30), Cefotaxime (30), Piperacillin (100), Ticarcillin (75), Piperacillin-Tazobactam (100/10), Ticarcillin-Clavulanic acid (75/10), Ceftazidime (30), Cefepime (30), Aztreonam (30), Imipenem (10), Meropenem (10), Ertapenem (10), Colistin (10), Gentamicin (10), Tobramycin (10), Amikacin (30), Netilmicin (30), Ciprofloxacin (5), Levofloxacin (5), Lomefloxacin (10), and Ofloxacin (5) (HiMedia Laboratories Pvt. Ltd., Mumbai, India).* P. aeruginosa* ATCC* 27853*,* E. coli* ATCC* 25922*,* E. coli* ATCC* 35218*, and* K. pneumoniae* ATCC* 700603* were used as quality control strains.

### 2.3. MIC Determination

Minimum inhibitory concentrations (MICs) of antibiotics were determined by VITEK-2 AST-GN25 and AST-GN280 susceptibility cards in accordance with CLSI recommendations and manufacturers' instructions, except tigecycline and colistin, for which the 2012 European Committee on Antimicrobial Susceptibility Testing break points were used [5, 6]. MICs were further determined by the *E*-test (bioMérieux, Marcy l'Etoile, France). According to Centers for Disease Control and Prevention (CDC), CRE are defined as Enterobacteriaceae that are nonsusceptible to penicillins, third-generation cephalosporins (ceftriaxone, cefotaxime, and ceftazidime), and one of the Carbapenems (doripenem, meropenem, and imipenem).

### 2.4. Phenotypic Screening for the Carbapenemase Production

Isolates with reduced susceptibility to meropenem and imipenem (diameter of zones of inhibition ≤13 mm) by disc diffusion method were screened for the production of carbapenemase. The phenotypic detection of the carbapenemase production was performed by the modified Hodge test by using ertapenem and meropenem discs (10 *μ*g) as per CLSI guidelines [5]. For MHT* K. pneumoniae* ATCC BAA-1705 and BAA-1706 were used as positive and negative controls, respectively. Metallo-beta-lactamase production detected by double-disc synergy tests (DDST) with both imipenem and meropenem discs (10 ug) plus disc containing (750 ug) of EDTA as described earlier by Lee et al. [7] and combined-disc synergy test (CDST) as described previously by Franklin et al. [8] by using imipenem/meropenem (10 *μ*g) discs and one disc with 292 *μ*g EDTA.* K. pneumoniae* ATCC BAA-2146 and* P. aeruginosa* ATCC 27853 were used as positive and negative controls, respectively. Ratio of MICs of imipenem (IP) to IP plus EDTA (IPI) was carried out using MBL (IP/IPI) *E*-test method as per manufacturer's instructions.

### 2.5. DNA Extraction and Molecular Detection

DNA was extracted from the bacterial isolates using the spin column method (QIAGEN; GmbH, Hilden, Germany) as per manufacturer's instructions. PCR-based detection of ESBL genes (*bla*_CTXM_, *bla*_SHV_, *bla*_TEM_, and *bla*_OXA_), Ambler class B MBLs (*bla*_IMP_, *bla*_VIM_, *bla*_SPM_, *bla*_GIM_, *bla*_SIM_, and *bla*_NDM-1_), Ambler class D (*bla*_OXA-23_, *bla*_OXA-24_, and *bla*_OXA-48_), and serine class A carbapenemases (*bla*_KPC_, *bla*_GES_, and *bla*_NMC_) were carried out on the isolates by using Gene Amp 9700 PCR System (Applied Biosystems, Singapore) [9–12]. PCR products were run on 1.5% agarose gel, stained with ethidium bromide visualized under UV light and photographed. The amplicons were purified using QIAquick PCR purification kit (QIAGEN; GmbH, Hilden, Germany).

### 2.6. DNA Sequencing and Sequence Analysis

Automated sequencing was performed on an ABI 3730XL DNA analyzer using the Big Dye system (Applied Biosystems Foster City, CA, USA). Sequences were compared with known sequences using the BLAST facility (http://blast.ncbi.nlm.nih.gov/).

### 2.7. Conjugation Experiments

Transfer of resistance genes by conjugation was assayed by mating experiments in Luria-Bertani broth using* Enterobacter* isolates (Parental strains) as donors and an azide-resistant* E. coli* J53 as the recipient strain using 1 : 10 ratio. The transconjugants were selected on Luria-Bertani agar with selection based on growth on agar in the presence of ceftazidime (30 *μ*g/mL) and sodium azide (100 *μ*g/mL). Plasmids were separated and compared by coelectrophoresis with plasmid of known sizes from* E. coli* (V517 and 39R861) on a horizontal 0.5% agarose gel at 50 volts for 3 hrs. Bands were visualized with UV transilluminator after staining with 0.05% ethidium bromide.

### 2.8. Strain Molecular Typing

Repetitive element based PCR (REP-PCR), Enterobacterial Repetitive Intergenic Consensus (ERIC-PCR), and Randomly Amplified Polymorphic DNA (RAPD) assays were performed to characterize* Enterobacter* strains recovered from patients [13, 14].

### 2.9. Plasmid Analysis

Plasmid from the parental strains and their transconjugants was extracted by using Qiagen plasmid mini kit (GmbH, Hilden, Germany) as per manufacturer's instructions. Extracted plasmid DNA was subjected to plasmid based replicon incompatibility (Inc) typing by using eighteen pairs of primers to perform five multiplex and three single PCRs which recognized F, FIA, FIB, FIC, B/O, X, Y, N, P, W, T, A/C, HI1, HI2, I1-Ic, L/M, K, and FII replicons as described previously [15]. Plasmid replicons were determined for the ESBL as well as carbapenemase producing clinical isolates.

## 3. Result and Discussion

A total of 70/130 (53.84%) isolates of* Enterobacter* spp. were found to exhibit reduced susceptibility to imipenem (diameter of zones of inhibition ≤13 mm) by disc diffusion method. Among 70 isolates tested, 48 (68.57%) isolates showed MIC values for imipenem and meropenem ranging from 32 to 64 mg/L as per CLSI breakpoints. Twenty-two, out of 70 isolates tested, showed MIC values below 8 mg/L. All of these 70 isolates were found susceptible to Colistin* in vitro* as per MIC breakpoints (<0.5 mg/L). Phenotypic characterization of* Enterobacter* spp. isolates (*N* = 130) from clinical samples is shown in Table 1. PCR carried out on these 48 MBL (IP/IPI) *E*-test positive isolates (12* Enterobacter aerogenes*, 31* Enterobacter cloacae*, and 05* Enterobacter cloacae* complex) was validated by sequencing for beta-lactam resistant genes and results were interpreted accordingly. Distribution of carbapenem resistant genes among* Enterobacter* spp. depicted in [Table 2]. VIM-2, VIM-6, and NDM-1 genes were found in carbapenem resistance isolates. Among ESBLs *bla*_TEM-1_, *bla*_SHV-28_, *bla*_SHV-12_, *bla*_CTX-M-15_, *bla*_OXA-10_, *bla*_OXA-9_, *bla*_OXA-2_, and *bla*_OXA-1_ were detected. These 48 isolates were further studied for conjugation assays and plasmid typing. Bacterial identification of the transconjugants from Luria-Bertani agar was performed by using VITEK-GNI cards and MICs of antibiotics were determined by VITEK-2 AST susceptibility cards. MICs values of ceftazidime (CAZ), ceftriaxone (CRO), cefepime (FEP), Piperacillin-Tazobactam (PIT), Piperacillin (PIP), Ticarcillin (TIC), Cefotaxime (CTX), Cefoxitin (FOX), imipenem (IMP), meropenem (MEM), and ertapenem (ETP) were high among transconjugants, whereas MICs of amikacin (AMK), gentamicin (GEN), tobramycin (TOB), ciprofloxacin (CIP), moxifloxacin (MXF), levofloxacin (LVX), tigecycline (TGC), and colistin (CST) fall within susceptible range. MICs of 42 clinical Enterobacter isolates recovered from Pus, Blood and urine along with their corresponding transconjugants were presented in the supplementary data file available online at http://dx.doi.org/10.1155/2014/972646. Conjugation experiments revealed that *bla*_NDM-1_ was transferable via a plasmid along with other beta-lactamase genes carried on other plasmids. Plasmid profiling of the isolates showed that *bla*_NDM-1_ was carried on plasmids ranging in sizes from 35 to 170 kb and *bla*_VIM_ was carried on 70 to 200 kb size plasmids.

### 3.1. Strain Molecular Typing

REP-PCR, ERIC-PCR, and RAPD assays as per banding pattern confirmed presence of eight, four, and three clones among* E. cloacae*_*(A–H)*_,* Enterobacter aerogenes*_*(A–D)*_, and* Enterobacter cloacae* complex_*(A–C)*_, respectively.* E. cloacae* strain typing showed 8 clones, among them three in blood, two in urine, and three in pus, respectively, while in case of* Enterobacter aerogenes*, two clones were detected in medical and two were in surgical wards.* Enterobacter cloacae* complex showed two different clonality in Medical ICU where as in surgical ICU isolates were from single clone.

### 3.2. Plasmid Replicon Typing

Plasmids purified from the clinical isolates were typed by PCR based replicon typing. IncFIA, IncFIB, IncFIC replicons were associated with *bla*_TEM-1_. Majority of *bla*_SHV_ showed association with multiple replicons (either IncFII, IncFIB or IncFIA, IncFIB); five isolates showed single replicon association (IncFIC). The *bla*_NDM-1_ gene in* Enterobacter* spp. was located on IncA/C, IncFII, and IncN plasmid. The *bla*_VIM_ was carried on plasmids belonging to IncP, IncW, IncFII, and IncFIB replicons. *Bla*_CTX-M-15_ was associated with multiple replicons of plasmid (IncFIA, IncFIB). The *bla*_OXA_ identified on plasmids was associated with IncP, IncHI2, IncFIC, and IncW replicons.* Enterobacter* infections can be acquired from exogenous as well as endogenous sources being ubiquitous in nature as a saprophyte in soil and sewage and as a commensal in human gastrointestinal tract. It is present in the feces of humans, animal excreta, dairy products, plants, plant materials, insects, and water [16–18]. Outbreaks of* Enterobacter* infection associated with contaminated intravenous solutions, blood products, distilled water, endoscopes, stethoscopes and other health care devices have been reported [19–22],* Enterobacter* infections in a health care settings, seems to arise endogenously from a previously colonized site in an infective individual, mainly the colonization of the gastrointestinal tract with* Enterobacter* spp. in the debilitated patients. Sometimes colonization of more than one strain is seen among those patients who already have been hospitalized and were on antibiotic therapy. Colonization leads to infection by this organism. Prolonged hospital stay, debilitating underlying illnesses, immunosurveillance and indewelling devices/implants have been risk factors for* Enterobacter* spp. infection in hospital settings [23].* E. cloacae* and* E. aerogenes* are the two most common* Enterobacter* species causing nosocomial infections, most frequently associated with disease. Antimicrobial resistance in* Enterobacter* strains varies with geographic locations. Whereas resistance to betalactam antibiotics, aminoglycosides, trimethoprim-sulfamethoxazole, and quinolones is more prevalent in southern Europe, Belgium, and Israel, in Greece, resistance to cefotaxime, ceftazidime, ceftriaxone, and aminoglycosides is prevalent in 60 to 70% of strains. 2–10% resistance to fluoroquinolones have been documented in various reports [24–30]. The emergence of AmpC, ESBL, and carbapenemase producers along with multiple resistant isolates poses a serious problem in the hospital settings. In our study, among* Enterobacter* spp. 25.71% (18/70) metallo-beta-lactamase production seen in blood stream infections, followed by 18.57% (13/70) surgical site infections, 15.71% (11/70) urinary tract infections, 8.57% (6/70) respiratory secretions. In 2010, CDC first reported carriage of NDM-1 in* E. cloacae* from patients who received medical care in India [31], following which various reports for the same were published by various authors. Khan and Nordmann reported presence of *bla*_NDM-1_ from cases of diabetic foot ulcer [32]. Lascols et al. and Castanheira et al. also reported carriage of *bla*_NDM-1_ among* E. cloacae* [33, 34]. Emergence of *bla*_NDM-1_ producing* E. cloacae* clinical isolates was reported from Singapore [35], China [36], Australia [37], United States [38], Kuwait [39], Turkey [40], and Canada [41]. MBLs other than NDM-1 also have been reported by various authors in* E. cloacae*: *bla*_IMP-1_ from Turkey [42], *bla*_IMP-8_ from Taiwan [43], *bla*_VIM1-4_ from Italy [44–46], *bla*_VIM-2_ from Korea [47], and *bla*_VIM-12_ from Greece [48]. In our study, we detected *bla*_VIM-2_ and *bla*_VIM-6_ among* Enterobacter* spp. Presence of *Bla*_OXA-48_ in* E. cloacae* have been reported in literature [49, 50]. However, our isolates were negative for OXA-48 like gene. Three studies from abroad by Brink et al. [51], Dai et al. [36], and Ageevets et al. [52] reported presence of *bla*_KPC-2_ in* E. cloacae*. Our study showed negative result for *bla*_KPC_. Carbapenems are one of the important antibiotics used to treat serious infections caused by Enterobacteriaceae. Multidrug resistance in Enterobacteriaceae is associated with significant morbidity and mortality. Therefore, it is important to check constantly the prevalence of resistance to carbapenem in Gram-negative organisms. Multidrug resistance due to the presence of MBL carrying genes is a point of concern as few drugs can be used for the treatment. The transfer of these genes through plasmids increases the spread of drug resistance from one species to another. Hence, early detection of these drug resistance genes by molecular methods is essential in limiting the spread of infection due to these organisms.

## Supplementary Material

MICs of 42 clinical Enterobacter isolates recovered from Pus, Blood and urine along with their corresponding transconjugants were presented in the supplementary data file.

## Figures and Tables

**Table 1:** Phenotypic characterization and distribution of *Enterobacter* spp. isolates (*N* = 130) from clinical samples.

Samples	Number of isolates	Carbapenem resistance by disc diffusion	MHT	CDST	DDST	MBL *E*-test
Urine	40	22	9	10	10	11
Blood	32	21	13	13	15	18
Pus	22	18	10	10	11	13
Sputum	8	1	1	1	1	1
Body fluids (synovial, pleural, and ascitic fluid)	10	0	0	0	0	0
Endotracheal	8	4	3	3	3	3
BAL	6	4	2	2	2	2
Tissue	4	0	0	0	0	0
Total	**130**	**70**	**38**	**39**	**42**	**48**

**Table 2:** Distribution of carbapenem resistance genes among *Enterobacter* spp. (*N* = 48).

Organism	VIM-2	VIM-6	NDM-1
*E. aerogenes* (*N* = 12)	4	2	6
*E. cloacae *(*N* = 31)	10	8	13
*E. cloacae* complex (*N* = 5)	4	1	0
